# Low Dose of Lipopolysaccharide Pretreatment Preventing Subsequent Endotoxin-Induced Uveitis Is Associated with PI3K/AKT Pathway

**DOI:** 10.1155/2017/1273940

**Published:** 2017-07-18

**Authors:** Nan Zhang, Shuo Yu, Xinli Liu, Hong Lu

**Affiliations:** Department of Ophthalmology, Beijing Chao Yang Hospital, Capital Medical University, No. 8 South Gongren Tiyuchang Road, Chao Yang District, Beijing 100020, China

## Abstract

**Purpose:**

To explore the effects of LPS pretreatment on endotoxin-induced uveitis and PI3K/AKT pathway.

**Methods:**

Endotoxin-induced uveitis was induced by a single subcutaneous injection of 200 *μ*g LPS. For the endotoxin tolerance group, induction of EIU was preceded by daily subcutaneous injection of 0.1 mg/kg LPS for five days. Clinical scores were graded at 24 h after EIU under a slit lamp microscope. HE stain was performed to observe the histopathology. Aqueous humor TNF-*α* concentration was quantified with enzyme-linked immunosorbent assay. The expressions of PI3K and AKT were detected through Western blot analyses, and the activation of AKT was detected through immunofluorescence study.

**Results:**

Endotoxin tolerance produced suppressive effects by significantly reducing the inflammatory reaction of anterior segment of the rats as measured by slit lamp and histopathology. Low dose of LPS pretreatment significantly reduced TNF-*α* concentrations and the expressions of PI3K and AKT. Furthermore, the activation of AKT was also inhibited.

**Conclusions:**

LPS pretreatment can ameliorate endotoxin-induced uveitis in rats. This protection of endotoxin tolerance against EIU is associated with PI3K/AKT pathway by reducing level of TNF-*α* in the aqueous humor.

## 1. Introduction

Uveitis is one of the world's major sight-threatening diseases, which mainly affects the iris, ciliary body, and choroid [1]. Lipopolysaccharide (LPS), which is a kind of pathogen-associated molecular patterns (PAMPs), activates the innate immune system through toll-like receptor 4 (TLR4) which is a member of innate immune recognition receptors (PRRs) [2]. Substantial clinical and experimental evidences support the role of LPS in the pathogenesis of immune-mediated, noninfectious acute anterior uveitis (AAU), particularly human leukocyte antigen- (HLA-) B27-associated AAU [3]. In terms of LPS-induced inflammatory response, there is an important protective mechanism, which is called endotoxin tolerance that the body or cells preexposed to low dose of LPS are refractory to subsequent LPS challenge [4]. Elucidating the effect of endotoxin tolerance on ocular inflammation and the underlying mechanism are of great importance for the prevention and treatment of uveitis.

Endotoxin-induced uveitis (EIU) with the injection of LPS into certain susceptible strains of rodents inducing an acute and preferential inflammation of the iris and ciliary body is a well-established animal model that closely resembles AAU in humans [5]. The inflammation of the cellular infiltration and protein leakage into the anterior chamber of the eye reaches a maximum 24 h after LPS injection [6]. Elevated expression of cytokines such as TNF-*α* and IL-6 has been observed to be concomitant with maximum EIU [6, 7]. Cytokine levels, especially those of TNF-*α*, can be used as a marker for monitoring the inflammation and disease progression.

As is known to us, LPS could activate the PI3K/AKT pathway, which regulates the expressions of cytokine and chemokine, such as TNF-*α* [8–10], moreover, which is involved in endotoxin tolerance [11]. Furthermore, a study of gene expression microarray analysis revealed that PI3K and AKT play critical roles in HLA-B27-associated AA [12]. We sought to explore the effect of endotoxin tolerance on endotoxin-induced uveitis and evaluate whether endotoxin tolerance could modulate the PI3K/AKT pathway.

## 2. Materials and Methods

### 2.1. Animals

Inbred male pathogen-free Wistar rats (8–10 weeks old, weighing 180–200 g) were obtained from the Vital River Laboratory Animal Technology Co. Ltd. (Beijing, China) and maintained in an air-conditioned room with 12 h light/12 h dark cycles. Food and water were supplied ad libitum. Experiments were conducted in accordance with the Institute for Laboratory Animal Research guidelines (Guide for the Care and Use of Laboratory Animals).

### 2.2. Reagents

Lipopolysaccharide (*V. cholerae*, classical Biotype, serotype Ogawa) was provided by the Lanzhou Biological Product Research. Rabbit polyclonal PI3-kinase p85*α* antibody and rabbit polyclonal AKT 1/2/3 antibody were purchased from Santa Cruz Biotechnology, CA, USA. AffiniPure Fab Fragment Donkey Anti-Rabbit IgG (H+L), Alexa Fluor® 488 were purchased from Jackson ImmunoResearch Laboratories, Inc., PA. ReverTra Ace qPCR RT Master Mix and SYBR Green Realtime PCR Master Mix were purchased from Toyobo (Shanghai) Biotech, Co., Ltd. Rat TNF-*α* ELISA kit was purchased from Cusabio.

### 2.3. Animal Model

Endotoxin-induced uveitis (EIU) was induced by a single subcutaneous injection of 200 *μ*g LPS dissolved in 0.1 ml sterile saline (1 mg/kg) as previously described [13]. All animals were randomly divided into three groups: normal control (NC) group, endotoxin tolerance (ET) group, and endotoxin-induced uveitis (EIU) group. In the ET group, endotoxin tolerance was induced by daily subcutaneous injection of 0.1 mg/kg LPS for five days [14]. The other two groups of rats were treated with sterilized saline in the same manner as pretreatment. On day 6, the animals in the ET and EIU groups received a single subcutaneous injection of 200 *μ*g LPS to induce EIU. The NC group received subcutaneous injection of 0.1 ml sterile saline.

### 2.4. Clinical Manifestations Scoring

Clinical features of ocular inflammation were evaluated in both eyes at 24 h after injection of 200 *μ*g LPS or vehicle under a slit lamp microscope. Inflammatory signs were recorded. The severity of uveitis was graded according to the scoring system in Table 1 by two observers who were blinded to the treatment groups [15, 16].

### 2.5. Enzyme-Linked Immunosorbent Assay (ELISA)

After clinical evaluation, an aqueous humor sample was collected from both eyes of all rats using a 30-gauge needle attached to a 1 mL syringe under a microscope. TNF-*α* levels in the aqueous humor were assessed with rat TNF-*α* ELISA kit. All measurements were conducted according to the manufacturer's instructions.

### 2.6. Histopathological Examination

For histopathologic analysis, the rats were killed 24 hours after the last injection of LPS or vehicle; their intact eyes were enucleated and placed in 10% neutral buffered formalin solution for 24 h. The eye specimens were dehydrated in a graded ethanol series and embedded in paraffin. Sagittal sections (4-5 *μ*m thick), cut near the optic nerve head, were stained with hematoxylin and eosin. As previously described [16, 17], anterior chamber tissues were scored for severity of inflammation as follows: grade 0 = normal tissue; grade 1+ = dilated iris vessels and thickened iris stroma with exudate, protein, and/or a few scattered inflammatory cells in the anterior chamber; grade 2+ = infiltration of inflammatory cells into the stroma of the iris and/or ciliary body with a moderate number of inflammatory cells within the anterior chamber; grade 3+ = heavy infiltration of inflammatory cells within the iris stroma, ciliary body, and the anterior chamber; and grade 4+ = heavy exudation of cells, dense protein aggregation in the anterior chamber, and in inflammatory cell deposits on the corneal endothelium. Histopathologic analyses were performed by a single pathologist blinded to treatment history.

### 2.7. Western Blot

Each ICB sample was harvested, rinsed with PBS, and lysed. A total of 40 *μ*g of protein per sample was separated by SDS-polyacrylamide gel electrophoresis, incubated with anti-PI3-kinase p85*α* (Santa Cruz Biotechnology) at a dilution of 1 : 1000 or anti-AKT1/2/3 (Santa Cruz Biotechnology) at a dilution of 1 : 500. Signal was visualized after secondary antibody staining (donkey anti-rabbit IgG antibody, 1 : 4000). All values were normalized with *β*-actin as loading control. Each sample was collected from two rats (4 eyes).

### 2.8. Immunofluorescence

Eye specimens were fixed in 4% paraformaldehyde for 1-2 h after being enucleated. The ICB was dissected into four segments; each prepared tissue was permeabilized with 0.3% Triton X-100 for 30 min at room temperature and then washed three times with PBS. After blocking nonspecific binding with 3% bovine serum albumin (BSA)/PBS, the tissues were incubated with rabbit polyclonal PI3-kinase p85*α* antibody (1 : 100 in 3% BSA/PBS) and rabbit polyclonal AKT 1/2/3 antibody (1 : 100 in 3% BSA/PBS), respectively, overnight at 4°C. Excessive antibodies were removed by washing with PBS, three times. The tissues were incubated with AffiniPure Fab Fragment Donkey Anti-Rabbit IgG (H + L), Alexa Fluor 488 (1 : 200 in PBS), for 2 h, protected from light at room temperature. After three washes with PBS for 10 min each, the slides were mounted in Antifade Solution. The negative controls were included replacing the first or second primary antibody or both antibodies with species- and isotype-matched irrelevant antibodies. Blank controls were included replacing the first or second primary antibody with PBS.

The slides were examined under a fluorescence microscope (Leica DM-4000B; Leica, Wetzlar, Germany). The images were captured, and the pairs of images were superimposed for colocalization analysis using image-management software (Adobe Photoshop CS3. 10.0; Adobe Systems, Mountain View, CA).

### 2.9. Statistical Analysis

Quantitative data were analyzed with one-way analysis of variance (ANOVA) (SPSS 19.0; SPSS Inc., Chicago, IL). Values of *p* < 0.05 were considered statistically significant.

## 3. Results

### 3.1. Endotoxin Tolerance Attenuates EIU

**Figure 1:**
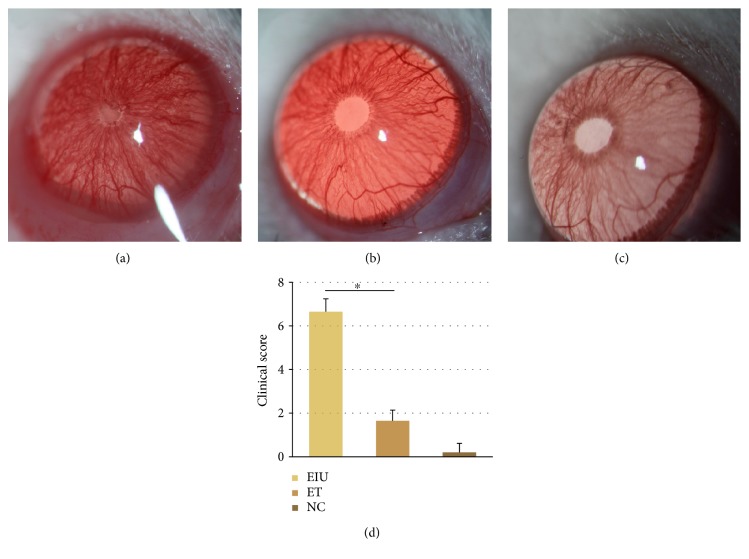
(a) Occlusion of pupil and fibrinous membrane was observed in the EIU group at 24 h after 200 *μ*g LPS injection. (b) Slight iris blood vessel dilatation could be found in the ET group at 24 h after 200 *μ*g LPS injection. (c) No ocular inflammatory signs were observed in rats of the NC group. (d) All data shown as mean ± standard deviation (*n* = 20, eye). ^∗^*p* < 0.05 significantly different from EIU group.

At 24 h after injection of 200 *μ*g LPS or vehicle, the EIU group showed typical signs mimicking human uveitis, including ciliary congestion, iris blood vessel dilatation, pupil occlusion, and fibrinous membrane formation (Figure 1(a)). Low dose of LPS pretreatment (ET group) significantly attenuated iris blood vessel dilatation, pupil occlusion, and fibrinous membrane formation; just slight iris blood vessel dilatation could be found in the ET group, small amounts of exudate occasionally (Figure 1(b)). No ocular inflammatory signs were observed in rats of the blank control group (Figure 1(c)). The clinical score of the ET group was 1.65 ± 0.49, significantly lower than that of the EIU group (6.65 ± 0.59, *p* < 0.05) (Figure 1(d)).

### 3.2. Histopathological Changes

**Figure 2:**
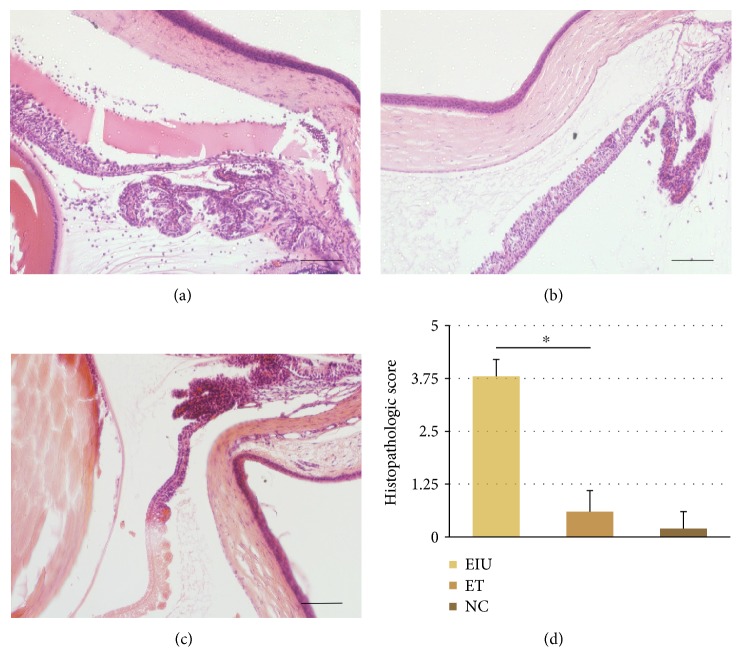
(a) The EIU group: heavy infiltration of inflammatory cells within the iris stroma, ciliary body, and the anterior chamber and inflammatory cells deposited on the corneal endothelium. (b) The ET group: only a few scattered inflammatory cells. (c) The NC group: no infiltrating cells. Scale bars: 200 *μ*m. (d) All data shown as mean ± standard deviation (*n* = 20, eye). ^∗^*p* < 0.05 significantly different from EIU group.

HE staining results were in accordance with clinical features of ocular inflammation. In the EIU group, heavy infiltration of inflammatory cells within the iris stroma, ciliary body, and the anterior chamber and inflammatory cells depositing on the corneal endothelium were observed (Figure 2(a)). Inflammatory cellular infiltration was significantly reduced by low dose of LPS pretreatment in the ET group (Figure 2(b)). The histopathologic score of the ET group was 0.6 ± 0.5, significantly lower than that of the EIU group (3.8 ± 0.4, *p* < 0.05) (Figure 1(d)).

### 3.3. The Levels of Cytokines in the Aqueous Humor during Endotoxin-Induced Uveitis in Wistar Rats

**Figure 3:**
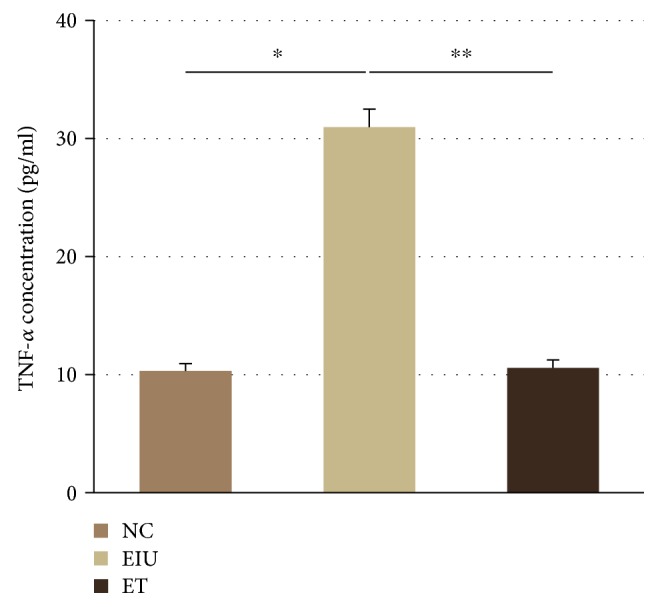
Expression of TNF-*α* in aqueous humor. All data shown as mean ± standard deviation (*n* = 10). ^∗^*p* < 0.05 the EIU group versus the NC group; ^∗∗^*p* < 0.05 the ET group versus the EIU group.

As shown in Figure 3, the aqueous TNF-*α* concentration in the EIU group (30.96 ± 1.55 pg/ml) was significantly higher than that of the NC group (10.32 ± 0.61 pg/ml). However, the level of TNF-*α* was significantly reduced in the ET group when dose of LPS pretreatment (10.58 ± 0.67 pg/ml) is low.

### 3.4. Effects of Endotoxin Tolerance on the Expression of PI3K and AKT in the Iris-Ciliary Body

**Figure 4:**
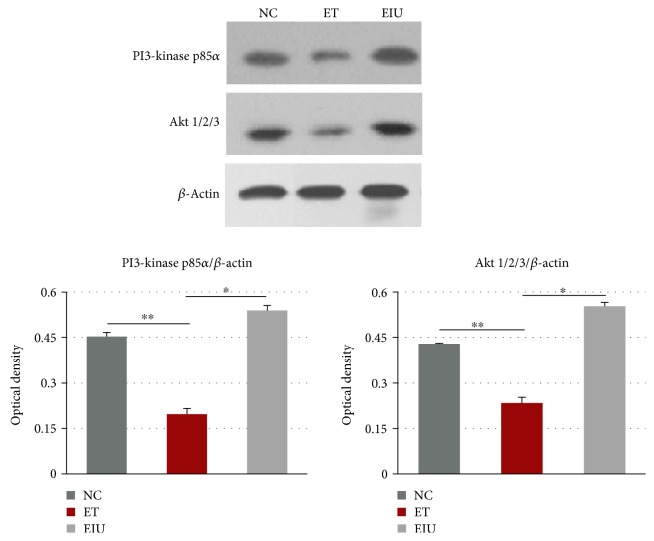
Western blot analysis and protein levels of PI3K and AKT. ^∗^*p* < 0.05 the ET group versus the EIU group; ^∗∗^*p* < 0.05 the ET group versus the NC group. Data represent the mean ± SD from ten independent experiments.

Western blot was performed to detect the expression of PI3K and AKT. As shown in Figure 4, the protein levels of PI3K and AKT in the ET group decreased significantly compared with the EIU group. Moreover, the levels also decreased significantly compared with the NC group.

### 3.5. Immunofluorescence Analysis of the AKT Activation

**Figure 5:**
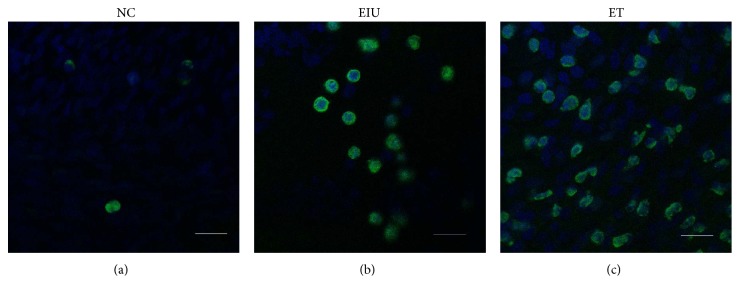
In the NC group, AKT distributed over cytoplasm (a). In the EIU group, AKT transferred from cytoplasm to cell membrane (b). In the ET group, translocation of AKT was inhibited partially (c). Scale bars: 50 *μ*m.

Without LPS stimulation, AKT distributed over cytoplasm (Figure 5(a)). After 200 *μ*g LPS stimulation, AKT transferred from cytoplasm to cell membrane, representing activation of AKT (Figure 5(b)). However, low dose of LPS pretreatment inhibited translocation of AKT partially (Figure 5(c)).

## 4. Discussion

As a potent inflammatory stimulant, high doses of LPS lead to systemic inflammatory response syndrome and death [18]. However, if the body was given a small dose of LPS stimulation in advance, the body showed low or no response to the subsequent large dose or lethal dose of LPS stimulation. This important protective mechanism is called endotoxin tolerance [4].

To elucidate the effect of endotoxin tolerance on ocular inflammation, endotoxin-induced uveitis with that a well-established animal model of AAU was used in this study. The Wistar rats in the EIU group showed typical signs mimicking human uveitis, including ciliary congestion, iris blood vessel dilatation, pupil occlusion, and fibrinous membrane formation. However, the inflammatory reaction of anterior segment of the rats significantly decreased in the ET group as measured by slit lamp assessment and histopathology. These results demonstrate that endotoxin tolerance which was induced by low dose of LPS pretreatment could successfully suppress the ocular inflammation in the EIU model.

Further, these findings suggest that endotoxin tolerance is of great potential in the treatment and prevention of uveitis. However, the molecular mechanisms underlying the induction of endotoxin tolerance are not yet fully understood. Hoekzema et al. [15] demonstrated that repeated injection of endotoxin resulted in no detectable IL-6 in the aqueous humor and the absence of uveitis. Mashimo et al. [19] indicated that continuous high expression of IL-10 in the eye plays significant role in the mechanism of endotoxin tolerance in a rat model of EIU. In this study, TNF-*α* levels in the aqueous humor during endotoxin-induced uveitis in rats were investigated to further clarify the anti-inflammatory effect of endotoxin tolerance and explore the role of cytokines in this protective mechanism. Consistent with the literatures [6, 20–22], elevated TNF-*α* was observed in the EIU group, and the TNF-*α* levels were significantly reduced by low dose of LPS pretreatment in ET group. This result strongly confirmed the anti-inflammatory effect of endotoxin tolerance, which is consistent with the slit lamp assessment and histopathology. Furthermore, the result suggests that TNF-*α* as well as IL-6 and IL-10 is closely involved in the protective mechanism of endotoxin tolerance. However, the upstream mechanism remains unclear. As mentioned earlier, the PI3K/AKT pathway plays an important role in regulating LPS-induced cytokine such as TNF-*α* expression. Therefore, we next evaluated whether endotoxin tolerance could modulate the PI3K/AKT pathway.

Similar to many protein kinases, AKT has a specific AH/PH domain which mediates the interaction between signaling molecules. PI3K generates phosphoinositide-3,4,5-triphosphate (PIP3) that can bind to the AH/PH domain of AKT in response to upstream signals, resulting in AKT translocating to the plasma membrane and being activated [23]. Thus, both the expressions of PI3K and AKT in ICB of the EIU rats and activation of AKT were detected. In this work, the protein levels of PI3K and AKT in the ET group decreased significantly compared with the EIU group, which were associated with the inflammatory outcome of the rats. Moreover, the levels in the ET group were even lower than those in the NC group, which indicated that the expression of PI3K and AKT was suppressed when dose of LPS pretreatment is low. Furthermore, according to the immunofluorescence, the activation of AKT was also suppressed in the ET group. Taken together, both low expressions of PI3K/AKT and inhibition of AKT activation contribute to the induction of endotoxin tolerance.

In summary, the present study demonstrates that low dose of LPS pretreatment could prevent subsequent endotoxin-induced uveitis by reducing the expression of TNF-*α* in the aqueous humor, and this protective effect of endotoxin tolerance is associated with PI3K/AKT pathway. Furthermore, in virtue of the critical roles of PI3K and AKT in HLA-B27-associated acute anterior uveitis, endotoxin tolerance holds promise for the prevention and treatment of this sight-threatening diseases.

## Figures and Tables

**Table 1:** Scoring system for clinical evaluation of uveitis.

Clinical signs	Grade of uveitis (score)
Iris hyperemia	
Absent	0
Mild	1
Moderate	2
Severe	3
Pupil	
Normal	0
Miosis	1
Exudate in anterior chamber	
Absent	0
Small	1
Large	2
Hypopyon	
Absent	0
Present	1
Maximum possible score	7
